# Root cause analysis of the challenges to sustain effectiveness of clinical pharmacist-driven antimicrobial stewardship in a Chinese Tertiary Hospital

**DOI:** 10.3389/fpubh.2026.1810297

**Published:** 2026-04-10

**Authors:** Zhenying Zhao, Yue Zhou, Chao Li, Jing Xu, Chenrui Xu, Hao Lu, Yuyang Gao, Baichuan Yu, Guiling Shi, Yongjie Zhao, Zimeng Li, Fengkun Yang, Po Ding

**Affiliations:** 1 Department of Pharmacy, Tianjin Union Medical Center, The First Affiliated Hospital of Nankai University, Tianjin, China; 2 Department of General Surgery, Tianjin Union Medical Center, The First Affiliated Hospital of Nankai University, Tianjin, China; 3 University of California, Berkeley, Berkeley, CA, United States; 4 Intemed Hospital Management & Development Centre, Beijing, China; 5 Department of Nursing, Tianjin Union Medical Center, The First Affiliated Hospital of Nankai University, Tianjin, China

**Keywords:** antimicrobial stewardship, clinical pharmacist, Delphi method, interview, root cause analysis

## Abstract

**Introduction:**

To optimize pharmacist engagement in antimicrobial stewardship (AMS), this study assessed general surgeons’ knowledge, practices, and perspectives on AMS and pharmacist interventions. Although antibiotic utilization density has decreased consistently over three years following the involvement of clinical pharmacists, challenges to AMS persist.

**Methods:**

This study employed a root cause analysis of interviews with general surgeons at a hospital in Tianjin. The interviews were informed by a questionnaire developed using the Delphi method.

**Results:**

The interview analysis identified four key barriers: limited surgeon awareness of AMS guidelines, complex antibiotic selection driven by disease severity, patient-specific clinical factors, and unresolved complex cases requiring tailored approaches. Surgeons proposed four strategic recommendations: (1) refining the AMS system systematically; (2) sustaining AMS outcomes through multifaceted efforts; (3) increasing the frequency of pharmacistsurgeon interactions; and (4) expanding pharmacists clinical roles beyond supervision and training.

**Discussion:**

The findings underscore the necessity of enhancing surgeon education on AMS guidelines and policies. Furthermore, increasing the frequency of pharmacistsurgeon collaboration would better support antibiotic selection, adjustment, drug sensitivity test interpretation, and access to specialized treatment information, thereby addressing the clinical complexities hindering the efficacy of AMS. The study also highlights critical pathways to strengthen system-level management strategies amid evolving health care demands.

## 1 Introduction

In the context of increasing concerns surrounding the indiscriminate use of antibacterial agents, the issues of bacterial resistance, antibiotic-related side effects (1), and secondary infections (2) have emerged as pressing challenges. The phenomenon of antimicrobial resistance (AMR) is particularly serious and has evolved into a global health problem (3). In clinical applications, the overuse of antibiotics continues to increase. The high frequency and intensity of utilization not only result in the consumption of valuable health care resources but also exacerbates the development of AMR, which inflicts various types of harm upon patients. The emergence of AMR resulted in 1.14 million deaths directly attributable to bacterial resistance and 4.71 million deaths associated with resistance globally in 2021. Moreover, a recent forecast analysis revealed that by 2050, the annual death toll from infections caused by AMR could reach up to 8.22 million individuals worldwide, potentially surpassing the mortality associated with cancer (4). This alarming trend underscores the urgent need for concerted efforts in antimicrobial stewardship (AMS) to combat the escalation of AMR.

Several developed countries have demonstrated an active stance in reducing antimicrobial drug usage and addressing the escalation of bacterial resistance, having initiated their efforts at an earlier moment. Therefore, these countries have achieved certain noteworthy outcomes. For example, as early as 2000, the UK initiated the first national action plan aimed at combating AMR, accompanied by a strategic framework of the AMS. These factors facilitated the execution and advancement of antibiotic stewardship programs across British hospitals (5). In this context, specialized antimicrobial pharmacists were empowered to lead clinical audits, monitor local prescribing patterns, and provide bedside expert guidance as key members of multidisciplinary stewardship teams (6). In China, the issue of antibiotic abuse is serious (7), especially in primary medical facilities nationwide, where the irrational use of antibiotics is more prevalent. Research has shown that the rate of surgical inpatient utilization of antibacterial agents is as high as 69.03% (8). Despite significant efforts in recent years to reduce antibiotic prescription rates, inappropriate antibiotic prescribing remains prevalent in China (9). Therefore, the problem of the irrational use of antibiotics and AMR has existed for a long time and has become an enduring challenge that needs to be solved (10). In response to these challenges, China has successively issued a series of policies and regulatory measures to strengthen the AMS. In 2004, the Ministry of Health of P. R. China introduced the “Guidelines for Antibacterial Use in Clinical Practice,” aiming to provide clear directions and regulations for the use of antibacterial drugs (11). In 2005, the “National Surveillance Network of Antimicrobial Clinical Use” (present name: Center for Antibacterial Surveillance) and the “China Antimicrobial Resistance Surveillance System” were established (12). In 2011, the Chinese government launched a special campaign to reorganize antimicrobial utilization policies in health care settings, establish mandatory administrative strategies for the rational use of antimicrobials, and develop inspection systems. The corresponding protocol emphasized that the target antibiotic utilization intensity of hospitalized patients in general hospitals should be less than 40 defined daily doses (DDDs) per 100 inpatient days (ID) (13, 14). Moreover, since 2012, a series of documents related to AMS norms and policies have been issued (15), which has further strengthened supervision and control over this field.

**Figure 1:**
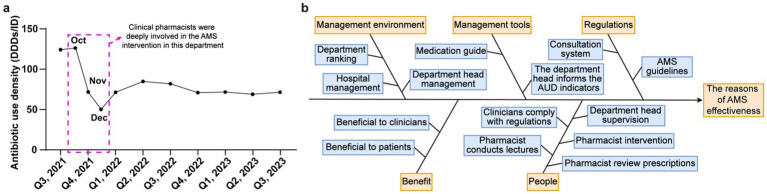
**(a)** Comparison of AUD before and after clinical pharmacist intervention in this general surgery department. **(b)** Analysis of the long-term maintenance of the AMS effectiveness in this general surgery department.

Currently, public hospitals in China are actively promoting clinical pharmacy services, including drug consultation, pharmaceutical ward rounds, medical advice auditing, and medication education, with the aim of increasing the safety, effectiveness, and cost efficiency of drug therapy (16). The professional practice of clinical pharmacists has been institutionalized through national mandates, notably the “Administrative Measures for the Clinical Use of Antibacterial Drugs” (2012). This regulation formally defines clinical pharmacists as mandatory members of the AMS organizational structure, tasking them with the authority to conduct prescription reviews, monitor antimicrobial usage intensity, and participate in multidisciplinary consultations (14, 16). Rather than being a localized hospital-level initiative, this integration is a part of national policy framework. Although certain achievements have been made, issues such as overuse and incorrect selection of antibacterial drugs remain. In particular, the consistently high DDDs/ID observed in the general surgery department of this hospital attracted specific attention from clinical pharmacists. Notably, prior to the formal commencement of this study, a substantial reduction in antibiotic usage density (AUD) had already been achieved in the general surgery department through the sustained interventions of clinical pharmacists (Figure 1a). This positive effect was maintained for 2 years preceding the present investigation. However, the potential for subtle fluctuations and the inherent complexity of clinical decision-making suggest that maintaining long-term efficacy remains an ongoing area for exploration. Building upon this foundation, the current study aimed to qualitatively assess surgeons’ knowledge and perspectives regarding AMS to identify the practical needs and challenges encountered in clinical practice. By exploring these underlying factors, we seek to develop more precise and sustainable intervention strategies for clinical pharmacists, thereby enhancing the stability of stewardship outcomes across various surgical departments.

## 2 Method

### 2.1 Study setting and participants

This study was conducted at a top-tier (Grade III) comprehensive hospital in Tianjin, China, with approximately 2,400 beds in total, 150 of which are designated for general surgery departments. The study included nine male surgeons from the general surgical ward (approximately 30% of the total surgeons in the Department of General Surgery), spanning various age groups between 30 and 60 years. Surgeons were specifically selected as study participants because they serve as the primary prescribers whose decision-making directly determines antibiotic utilization patterns. In addition to serving as trainers, these clinical pharmacists serve as stewards of AMS and acted as the initiators of the research interviews. Prior to the study’s commencement, a long-term professional relationship existed between the researchers and the participants through routine AMS activities, pharmaceutical ward rounds, and training sessions. This established rapport facilitated open communication during the interviews, although the researchers maintained a neutral stance to minimize bias. All participants had undergone a training program on the rational use of antibiotics organized by the hospital’s clinical pharmacist in 2021. The training comprised 6 centralized sessions scheduled across working days over 2 months, delivered at a rate of approximately one to two training sessions per week. The training was held annually, once each year, for this general surgery department in 2022 and 2023, with largely consistent participation across both years. The trainers are clinical pharmacists who have participated in clinical pharmacist training and instructor training organized by the China Hospital Association’s Pharmaceutical Affairs Professional Committee and have passed the assessment to obtain the relevant certification.

### 2.2 Data collection and analysis

The participant recruitment was formally initiated in October 2023, followed by face-to-face interviews conducted in November 2023. Prior to these interviews, all participating surgeons were provided with comprehensive information regarding the study and were required to sign a participant consent form in person. The participants also granted explicit permission for the researchers to record the conversations to ensure accurate data transcription. Furthermore, professional and demographic characteristics—including gender identity, professional title, educational background, and years of experience—were systematically recorded (see Supplementary Table S1).

The research process was initiated with the development of a structured assessment tool through a two-stage approach. First, the Delphi method was specifically employed to design an initial 20-item questionnaire (Appendix 1). This preliminary instrument was then evaluated in pilot interviews conducted with two directors from the general surgery department, who participated as interviewees to assess the tool’s clinical relevance and clarity. The primary aim of these questions was to explore the factors contributing to the long-term effectiveness of AMS implementation in hospitals. On the basis of the content and key factors discussed by the surgeons across the preliminary interviews, the questions were refined and condensed into a finalized questionnaire with 9 questions (Appendix 2).

The study was conducted in accordance with the principles and guidelines outlined in the Declaration of Helsinki. Building upon the refined questionnaire, the second round of interviews occurred approximately 1 month after the initial recruitment. One clinical pharmacist assumed primary responsibility for posing questions and guiding the conversation, whereas the assistant pharmacist focused on real-time documentation. Following the conclusions of all the interviews, the content analysis of the data was independently conducted by a second clinical pharmacist to ensure methodological rigor. Root cause analysis was used to identify the reasons for the sustained effectiveness of AMS on the basis of the findings. All potential factors were categorized and visualized using a fishbone diagram—a tool that employs “classification–stratification–visualization” logic to transform qualitative insights into a structured cause-and-effect map.

Finally, the analysis results, along with the interview content, provided a comprehensive understanding of the reasons behind the sustained effectiveness of AMS and the present challenges. To ensure analytical rigor, all clinical pharmacists conducting the analysis were blinded to participant identities. Antibiotic usage density data were obtained from the Hospital Information System of the hospital, with collection initiated in the third quarter of 2021. The data collection was carried out on December 1st, 2023. An analysis incorporating the COVID-19 epidemiological situation in Tianjin during the study period indicated that pandemic-related factors did not significantly influence antimicrobial utilization patterns.

## 3 Results

### 3.1 The long-term maintenance of AMS achievements is determined by multiple factors

On the basis of the AUD monitoring data from this general surgery department, following the in-depth participation of the clinical pharmacist in AMS work (Q4, 2021), a significant decrease in usage intensity was observed and sustained over a two-year period (Figure 1a). However, a slight rebound in usage intensity was noted after the first quarter of 2022. To investigate the factors underlying these fluctuations and identify the root factors impacting the sustainability of the AMS program effectiveness, we utilized the fishbone diagram framework (Figure 1b) to comprehensively present the possible cause–effect.

Moreover, surgeons proposed four strategies to maintain long-term achievements in AMS: (1) providing feedback to the clinical pharmacist about any special circumstances encountered once a month or quarter and conducting internal department learning sessions; (2) when facing special patients, surgeons should communicate and consult with clinical pharmacists in a timely manner to select more appropriate antimicrobial drugs; (3) organizing additional lectures and training sessions by clinical pharmacists to deepen surgeons’ understanding of AMS practices; and (4) following termly clinical pharmaceutical rounds and prescription reviews, the clinical pharmacists engage in one-on-one discussions with doctors who have issues with their antimicrobial prescriptions, thoroughly communicating prescription issues, alternative medication plans, and reasons.

### 3.2 Multiple factors affect AMS implementation

Qualitative analysis of the interviews revealed that several factors can impede the AMS interventions in actual clinical settings. For example, some surgeons mentioned their inadequate understanding of the contents and significance of AMS; some surgeons highlighted situations where they tended to opt for empirical medication as the initial step in treating complex infections during surgical diagnoses rather than prioritizing the AMS guidelines. This section reports on the four primary factors influencing AMS processes after the interview findings are categorized and analyzed.

#### 3.2.1 Surgeons lack awareness of AMS

The interviews revealed that surgeons could not completely articulate the requirements of national AMS policies. Instead, they were trying to combine known partial AMS guidelines with clinical experience to guess what they did not know about policies. The data illustrate the phenomenon of limited understanding among surgeons regarding the specifics of AMS practices and relevant national policies.

According to the feedback gathered from surgeons during interviews, they all believed that the AMS is necessary for clinical medication and treatment. Furthermore, surgeons highly valued the role of pharmacists in clinical medication and treatment and recognized their interventions as beneficial. They also acknowledged the positive outcomes achieved through current interventions of AMS. Meanwhile, some surgeons have suggested that the frequency of training about AMS, which is currently held annually, may not be aligned with the demands of clinical practice. Moreover, surgeons reported a preference for increased training frequency (e.g., monthly/quarterly) over the current annual AMS sessions.

“I think regular training in the management of antibacterial drugs is very important, especially for young physicians.” [Surgeon 5]

“I also want to emphasize the necessity of increased interaction between pharmacists and clinical practice. Clinical pharmacists should engage more with doctors to understand the realities of disease treatment and department-specific characteristics. In return, we should learn from clinical pharmacists through lectures and research activities to enhance our knowledge about AMS. These interactions, preferably once a month or quarterly, will do good to both sides.” [Surgeon 5]

“Clinical pharmacists should provide regular lectures on AMS guidelines and usage regulations of antibacterial drugs every two to three months. This thing should be available to all the hospital staff who are interested in taking part.” [Surgeon 6]

#### 3.2.2 Antibiotic selection contexts and pathways are complex

In clinical treatments, various common diseases necessitate the administration of antibiotics, including abdominal infection, appendicitis, biliary tract infection, enteritis infection, and surgical prophylaxis. In the interviews, some surgeons tended to resort to broad-spectrum antibiotics such as cephalosporins, β-lactamase inhibitors, and carbapenems for the treatment of critical patients. Among these, cefoperazone sodium, sulbactam sodium, and imipenem cilastatin sodium are commonly used in this general surgery department. In cases where the patient’s condition was severe, a subset of surgeons opted to utilize high-level antibacterial drugs directly and prolong the administration course (44.4%, Table 1), resulting in increased antibacterial drug utilization. Similarly, some surgeons reported prioritizing disease cure in extraordinary or unusual circumstances over strict adherence to guidelines about frequency and intensity of antibacterial drug usage (22.2%), for example, in cases of severe infection or multiple complications or severe medical conditions, such as shock and gastrointestinal perforation.

**Table 1:** The surgeon’s attitude toward the application of antibiotics under unusual circumstances (each person can only choose one option).

Items	Using broad-spectrum antibiotics	Using high-level antibiotics	Prioritizing disease resolution
Number of people	3	4	2

“I commonly use cephalosporins or metronidazole to treat appendicitis. Piperacillin sodium and tazobactam are the primary choices for biliary tract infections. Severe infections may require treatment with cefoperazone sodium and sulbactam sodium, imipenem and cilastatin sodium, and carbapenem antibiotics. Intense abdominal infections require a combination therapy or empirical approach to medication, such as the incorporation of vancomycin.” [Surgeon 1]

“In our department, antibiotics are predominantly utilized in treating abdominal infections, cholecystitis, appendicitis, acute intestinal obstruction, and acute abdomen. The intensity and quantity of antibiotic application are relatively minimal during selective operation, due to the standard preventive measures applied with us. However, in cases of preoperative infections, we were used to using therapeutic antibiotics directly. Additionally, for patients in severe conditions, more potent antibacterial medications or antibiotics with restricted-use levels were prescribed.” [Surgeon 5]

#### 3.2.3 Clinical factors affect antibacterial drug usage

In the process of choosing antibacterial drugs for clinical treatment, surgeons are influenced by various individual factors from patients. Primary considerations include the degree of severity, age, antibiotic efficiency, damage caused by adverse reactions to various therapeutic drugs, liver and kidney function, and even the patient’s economic status and mental state (Table 2). Following this, the selection of the appropriate antibiotic is based on the etiological foundation. Ultimate adjustments to antibiotic dosing and intensity involve patient liver and kidney function.

**Table 2:** The factors that surgeons need to consider during the treatment (each person can choose multiple options).

Items	Degree of severity	Age	Antibiotic efficacy	Adverse reaction	Economic status/mental state	Liver and kidney function
Number of people	6	4	4	9	3	9

“I think the importance of selecting the appropriate antibiotic by first considering the patient's condition. Factors such as the patient’s age, overall health, as well as liver and kidney functions, are also crucial in this decision-making process. For example, adjusting antibiotic dosage for patients with liver and kidney function abnormalities to minimize potential side effects.” [Surgeon 1]

“We usually prioritize the assessment of antibiotic efficacy, followed by an evaluation of potential side effects and adverse reactions. Additionally, factors such as the patient's age and economic status are also what we take into account.” [Surgeon 4]

“First, we need a comprehensive evaluation, involving diagnosing the presence of infections based on the patient's condition, considering pathogenic detection results, and taking into account all damage to liver and kidney function as well as potential side effects. For example, imipenem-cilastatin sodium causes the risk of mental disorders in some patients. It is also very important to adjust the dosage and frequency of antibiotic administration, especially after the infection is controlled. Moreover, we also need to evaluate the efficacy of the antibiotic after treatment and timely adjust the usage level of the antibiotic based on the infection control status: upgrade or downgrade the level.” [Surgeon 5]

#### 3.2.4 Several structural barriers pose difficulty in resolution

In clinical treatment, surgeons encounter various challenges when administering antibacterial drugs. These challenges include managing difficult and complicated diseases, addressing mixed and multisite infections, combating the emergence of drug-resistant bacteria, encountering infections with uncertain pathogens, investigating unexplained fevers, and preventing potential nosocomial infections (17–20). These complexities are associated with variations in surgeons’ decision-making processes concerning the selection of antibiotics, the dosage and frequency of antibiotic administration, and the potential need for antibiotic replacement. Moreover, these specific situations occur alongside high antibiotic usage prevalence and nonstandard intensity in clinical settings.

Some surgeons mentioned difficulties in drug-sensitive test interpretation and bacterial culture, which could influence AMS. They responded with some problems in drug-sensitive test results, such as unclear numerical units, lack of reference values, and inconsistent and conflicting phenomena between the outcomes of drug-sensitive tests and empirical diagnostic results. These problems affected their decision to change antibiotics or adjust dosages. Additionally, in terms of bacterial culture, insufficient clinical sample collection, inappropriate culture conditions, and suboptimal technical procedures may result in negative bacterial culture findings, drug use inconsistent with culture results and clinical symptoms, and prolonged culture duration. These factors are associated with variations in antimicrobial utilization patterns. Surgeons frequently encounter these challenges and often turn to other physicians from departments such as infectious immunology and respiratory medicine, as well as clinical pharmacists, for group consultation. Nevertheless, these complex issues persisted during the study period (Table 3).

**Table 3:** The complex problems that pose difficulty in resolution (each person can choose multiple options).

Items	Patient factors	Drug-sensitive test problems	Bacterial culture issues	Result distortion
Number of people	4	3	3	2

“When prescribing antibiotics, there were few challenges in treating common diseases, but like abdominal infection cases or antibiotic-resistant present cases immersed difficulty in antibiotic selection. Patients with mixed and multi-site infections also complicate the decision-making process. In general surgery, we sometimes face a tricky situation where patients have a fever with unexplained cause. This makes it tough to pinpoint exactly where the infection is coming from and which antibiotics to use to fight it.” [Surgeon 1]

“When adjusting the antibiotic treatment plan, uncertainty about what's causing the infection can really throw a wrench in things. Take fevers, for example. It is sometimes unclear whether it is a result of fungi, bacteria, or other pathogens.” [Surgeon 3]

“There are many issues worthy of attention. For example, in the results of the drug sensitivity test report, units seem to be prone to confusion. Some drug sensitivity test results were expressed as sensitive (S), intermediate (I), and resistant (R), while for others, it may be expressed by the minimum inhibitory concentration (MIC). And you know what? It's really annoying that there's no reference value. Also, some patients don't get better even after using antibiotics for about two weeks, but the drug sensitivity test result says it's sensitive!” [Surgeon 4]

“The bacterial culture doesn't always give accurate results. There are some kinds of pathogenic bacteria, but the culture testing often can't find them, even after trying a bunch of times. And on top of that, inaccurate drug sensitivity test reports were also a big deal. For example, you can see this when a drug sensitivity test report says a specific antibiotic should work, but the patient doesn't get any better after using it for three weeks. As a result, symptoms like fever and abdominal infections caused by pathogenic bacteria might just keep going on.” [Surgeon 6]

“First, the time for culturing pathogenic bacteria is often very long. Second, I suggest that all the commonly used antibiotics in the hospital should be included in the drug sensitivity test report. This is really important because a lot of the drugs listed in the report might not be available in our hospital, and that influences us to make timely and appropriate treatment options for patients.” [Surgeon 7]

### 3.3 The AMS system needs to be improved

Although the AMS work has achieved some successes, there are some areas that require improvement. According to the interviews, some surgeons did not comprehend the assessment system for the application of AMS. Moreover, a few surgeons only know the ranking feedback of the antibiotic utilization rate in different departments. Interestingly, five surgeons did not feel pressured by the current assessment system of AMS. Conversely, three surgeons expressed that the assessment system induced pressure, making them reluctant to prescribe antibiotics freely. Moreover, three surgeons emphasized that curing a patient’s illness outweighs controlling the utilization rate of antibiotics. The pressure stemming from AMS influences their decision-making regarding medication. Furthermore, some surgeons have suggested that examining the rationality of antibiotic use should be based on analyzing the actual circumstances from both physicians’ and patients’ perspectives, rather than just the AMS policies.

In the interviews, one surgeon gave a clear opinion on the AMS work: “From the perspective of a department director, the existing standards for AMS were deemed inadequate. This could lead to situations where young surgeons may hesitate to prescribe high-intensity antibiotics or even refrain from their use altogether owing to concerns about adhering to the guidelines concerning the utilization rate and intensity. As a consequence, patients may experience deterioration. Nevertheless, this issue has a minimal impact on surgeons with senior professional titles or high positions.

### 3.4 Clinical pharmacists should play an increased role in clinical practice, involving not only supervision and training

During the interviews, surgeons expressed their belief that clinical pharmacists play a crucial role in the AMS program and have shown positive outcomes. In addition, surgeons highlighted the contributions of clinical pharmacists in various other areas, such as promoting medication standardization, rectifying prevalent issues in daily diagnosis and treatment practices, and facilitating the incorporation of new antibacterial medications. Two surgeons proposed that clinical pharmacists should primarily undertake the responsibility of determining which antibiotics to administer, although under the current Chinese medical system, pharmacists do not have prescribing rights. However, they can be incorporated into the multidisciplinary treatment (MDT) process to support surgeons in making informed decisions regarding drug selection. Furthermore, a surgeon highlighted the importance of involving clinical pharmacists in diagnosing and supported the collaboration of pharmacists and doctors to create personalized medication plans.

“The sustained effectiveness of AUD is primarily attributed to the continuous and inspiring efforts of pharmacists team. We think your work is not a temporary task, but a long-term and ongoing responsibility. From a management perspective, the monthly publication of relevant indicators by your clinical pharmacists and our department director in the work chat group, is a crucial for the rational use of antibiotics. This consistent focus, extending from hospital leaders to department leaders and the diligent work of your clinical pharmacists, continually motivates us to maintain AUD effectively.” [Surgeon 5]

## 4 Discussion and conclusion

In actual clinical practice, complex situations often complicate antibiotic decision-making, potentially increasing the rate and intensity of their utilization (21). Various factors that affect surgeons contribute to difficulties in addressing issues related to the awareness of AMS and bacterial resistance (22). This study not only demonstrated the positive effectiveness of clinical pharmacists’ participation in AMS in the general surgery department but also further investigated the reasons why the effects of AMS lasted for more than 2 years through qualitative research interviews and root cause analysis. A key finding was the surgeons’ reported lack of systematic comprehension regarding AMS specifics and policies, identified as a significant contributor to the prevalent high rates and inappropriate usage of antibacterial drugs (23). This underscores a fundamental challenge and emphasizes the need for enhanced training and lectures on related AMS guidelines, policies, and regulations.

Crucially, our findings directly reflect surgeons’ perspectives on what sustains impact: surgeons explicitly attributed sustained AUD effectiveness primarily to two key pharmacist-driven activities: (1) ongoing training that equipped them with essential knowledge (e.g., indications and consultation), effectively supporting positive trends; and (2) consistent supervision and reminders facilitated by regular sharing of AUD data within communication platforms, which they deemed crucial for maintaining effectiveness. This concrete attribution underscores the value surgeons place on these specific, sustained pharmacist engagements. Furthermore, to stabilize the observed AUD metrics and address fluctuations, surgeons have proposed specific maintenance strategies aimed at fostering regular communication, consultation, and learning. Moreover, increasing the frequency of clinical pharmacist consultations and interactions is crucial. This will assist surgeons in selecting and adjusting antibacterial drugs efficiently, interpreting drug-sensitive test reports, and acquiring timely, efficient drug information when treating complex patients (24).

The sustained AUD reduction in our study aligns with aligns with international evidence demonstrating the efficacy of clinical pharmacist-driven AMS. Similar pharmacist-led successes are documented in the UK’s ‘Start Smart’ initiative and Japanese surgical departments (6). Furthermore, a Cochrane review which analyzed 131 studies globally, concluded that both restrictive and persuasive interventions are highly effective in reducing antibiotic treatment duration and usage density (25). Notably, the barriers identified in our analysis, including diagnostic uncertainty and clinician autonomy, are not unique to the Chinese context. These issues mirror well-documented international challenges, suggesting that addressing behavioral and structural complexities is essential for the long-term sustainability of AMS initiatives globally.

The interview analysis concurrently revealed underlying systemic barriers that require further characterization and targeted solutions. Moreover, some physicians perceive the current antibacterial drug management system as unreasonable, primarily because patient care often takes precedence over the constraints imposed by existing management systems. Consequently, the strict control of antibiotic utilization rates within standard parameters is not always feasible. This underscores the need for a more scientifically optimized AMS policy. However, in this context, clinical pharmacists have emerged as potential key players. Traditionally responsible for overseeing and instructing clinical medical personnel on medication usage, clinical pharmacists in China are increasingly assuming a more substantial role because of the gradual introduction of fees for pharmaceutical care in public hospitals. Their unique ability to collaborate with other health care professionals, such as surgeons and nurses, enables them to assist in optimizing medication therapy. Their in-depth understanding of drugs, including interactions, side effects, and patient tolerance, allow them to provide invaluable insights and decision support to doctors. Therefore, enhancing the intensity and frequency of clinical pharmacist interventions is vital to improve surgeons’ proficiency with AMS regulations and guidelines, thereby fostering an environment conducive to proposing constructive suggestions. Integrating clinical pharmacists more fully into the health care team remains essential for improving antibacterial drug management. Moreover, future initiatives will inevitably focus on improving guidelines for antimicrobial use.

This study also has several limitations. The interviews focused exclusively on the experiences of nine general surgery surgeons at a hospital in Tianjin, China. Therefore, the findings may not be applicable to other settings. The interviews highlighted the various challenges associated with antibiotic use that surgeons encountered and the effectiveness of interventions by clinical pharmacists. However, the impact weights of these challenges are uncertain, and whether clinical pharmacists’ interventions can be effective when multiple challenges are combined also remain unclear. Further research is needed to understand how AMS interventions are experienced across different contexts and departments to better understand the potential of clinical pharmacists in AMS.

## Data Availability

The raw data supporting the conclusions of this article will be made available by the authors, without undue reservation.
