# Supplementary material for: Root cause analysis of the challenges to sustain effectiveness of clinical pharmacist-driven antimicrobial stewardship in a Chinese Tertiary Hospital
Source: Front Public Health. 2026 Apr 10;14:1810297. doi: 10.3389/fpubh.2026.1810297 (PMC13106327; doi:10.3389/fpubh.2026.1810297)
Supplement: Supplementary file 1 [file Data_Sheet_1.docx]

**Supplementary Materials for**

**Root Cause Analysis of the Challenges to Sustain Effectiveness of Clinical Pharmacist-Driven Antimicrobial Stewardship in a Chinese Tertiary Hospital**

Zhenying Zhao^1†^, Yue Zhou^1†^, Chao Li^2^, Jing Xu^2^, Chenrui Xu^3^, Hao Lu^4^, Yuyang Gao^1^, Baichuan Yu^1^, Guiling Shi^1^, Yongjie Zhao^2^, Zimeng Li^5^, Fengkun Yang^1*^, Po Ding^2*^

^1^Department of Pharmacy, Tianjin Union Medical Center, The First Affiliated Hospital of Nankai University, Tianjin, China.

^2^Department of General Surgery, Tianjin Union Medical Center, The First Affiliated Hospital of Nankai University, Tianjin, China.

^3^University of California, Berkeley, USA

^4^Intemed Hospital Management & Development Centre, Beijing, China.

^5^Department of Nursing, Tianjin Union Medical Center, The First Affiliated Hospital of Nankai University, Tianjin, China.

^*^Corresponding author Email address:

13802107898@163.com (Po Ding)

[yangfengkun018@163.com](mailto:yangfengkun018@163.com) (Fengkun yang)

**Table SI**. Demographic and Professional Characteristics of the Interviewed Surgeons (N = 9).

| Characteristic | Category | Number of People | Percentage (%) |
| --- | --- | --- | --- |
| Working Years  (Mean: 20.13 years) | <10 years | 4 | 44.40% |
|  | 10–20 years | 3 | 33.30% |
|  | 30–40 years | 1 | 11.10% |
|  | >50 years | 1 | 11.10% |
| Education Level | Associate degree | 1 | 11.10% |
|  | Bachelor’s degree | 3 | 33.30% |
|  | Master’s degree | 3 | 33.30% |
|  | Doctorate degree | 2 | 22.20% |
| Professional Title | Resident physician | 2 | 22.20% |
|  | Attending physician | 1 | 11.10% |
|  | Associate chief physician | 4 | 44.40% |
|  | Chief physician | 2 | 22.20% |
| Administrative Position | Department director | 1 | 11.10% |
|  | Team leader | 2 | 22.20% |
|  | None | 6 | 66.70% |

**Appendix 1. Interview topic guide**

The following questions included in the questionnaire acted as a topic guide for the doctors. They were encouraged to voice their perspectives candidly, as there were no predetermined correct findings or incorrect answers. Prior to engaging with the survey, the participants provided their consent by signing a permission statement.

**Questions:**

1. Based on your understanding, could you detail the contents of antimicrobial stewardship (AMS)?"

2. Could you share your knowledge and understanding of the national policies pertaining to the AMS?

3. In your view, how does the management of antimicrobial drugs influence clinical practice?

4. In your career as a general surgeon, what are the typical scenarios where you prescribe or utilize antimicrobial drugs?

5. What are the key factors you consider when deciding on the use of antimicrobial drugs?

6. In the context of antibacterial drug application, what clinical scenarios do you find particularly challenging? Do you need the assistance from other medical professionals to address these issues?

7. What measures will you take if certain types of antibacterial drugs are in short supply?

8. Do you modify treatment plans based on drug sensitivity test outcomes following empirical medication? What challenges do you face when interpreting the drug sensitivity reports and selecting appropriate medications? Do you need the assistance of other medical professionals?

9. How do you evaluate the effectiveness of antibacterial drugs? When do you conduct such evaluations?

10. Based on your experience, what factors may impede AMS?

11. What factors in your department do you believe contribute to the successful implementation of the antibiotic management plan?

12. In the various continuing education programs conducted by clinical pharmacists, which topics have been most beneficial to your work? How specifically does it help?

13. How serious do you think the current issue of antibiotic resistance is? Do you consider bacterial resistance in your clinical decision-making?

14. What is your assessment of the effectiveness of AMS? What roles did the pharmacists play in the process, and what contributions did they make to the hospital or department?

15. Are you aware of the specific antibiotic use density (AUD) management goals set by your department or hospital? Do you understand the changes in AUD indicators before and after the implementation of management?

16. How often do you expect pharmacists to maintain intervention frequency in their future work?

17. Has the hospital's AMS system imposed significant pressure on your routine medication practices?

18. What do you think of the current antibiotic clinical application management supervision system of the hospital?

19. In the use of antibiotics, which kind of specific support or assistance would you appreciate from pharmacists?

20. Is there any additional information you would like to add or emphasize?

**Appendix 2. Finalized interview questionnaire**

1. When do you prescribe antibacterial drugs?

2. What factors do you consider when using antibacterial drugs?

3. Do you face difficulties in the use of antibiotics?

4. What assistance do you require when facing challenges with using antibiotics?

5. When and how should the efficacy of antibacterial drugs be evaluated?

6. What factors affect the AMS did you think? What do you believe are the reasons for the sustained effectiveness of our AMS program?

7. Do you believe that the current situation of bacterial resistance is serious?

8. What is your opinion on the significance of clinical pharmacists, and what recommendations do you have for them?

9. What are your opinions or recommendations on AMS?
